# 20-Hydroxyecdysone Mediates Reproductive Diapause in *Galeruca daurica* via Ecdysone Receptor *EcR* and Nuclear Hormone Receptor *HR3*

**DOI:** 10.3390/ijms252312976

**Published:** 2024-12-03

**Authors:** Ling Li, Zhihan Yao, Baoping Pang, Yanyan Li

**Affiliations:** Research Center for Grassland Entomology, Inner Mongolia Agricultural University, Hohhot 010020, China; lling@imau.edu.cn (L.L.); 15334730166@163.com (Z.Y.); pangbp@imau.edu.cn (B.P.)

**Keywords:** *Galeruca daurica*, 20-hydroxyecdysone, ecdysone receptor, nuclear hormone receptor, reproductive diapause

## Abstract

20-hydroxyecdysone (20E) signaling plays an important role in regulating insect growth, development, and reproduction. However, the effect of 20E on reproductive diapause and its regulatory mechanisms have not been fully understood. *Galeruca daurica* is a new pest in the Inner Mongolia grasslands, and it aestivates in an obligatory reproductive diapause form. In this study, the complete open reading frame (ORF) sequence of the *ecdysone receptor* (*EcR*) was cloned from *G. daurica*. Application of 20E promoted the expression of *EcR*, *nuclear hormone receptor* (*HR3*), and *vitellogenin* (*Vg*), whereas it reduced the expression of *fatty acid synthase* (*FAS*) and lipid content, leading to delayed diapause entry in female adults. Silencing *EcR* or *HR3* by RNAi increased the expression of *FAS* and lipid content, whereas it reduced the expression of *Vg*, inducing reproductive diapause. These results indicate that 20E may mediate reproductive diapause via a conserved EcR/HR3 cascade in *G. daurica*.

## 1. Introduction

Diapause is a genetically regulated program of arrested development that evolved as a survival strategy to avert environmental conditions unfavorable to continued development, activity, or reproduction. Adult diapause, also known as reproductive diapause, is characterized by arrested ovarian development, increased lipid accumulation, and reduced metabolism [1]. An increasing number of evidence has indicated that the absence of juvenile hormone (JH) induces reproductive diapause [2], and the JH signaling pathway is central to the regulation of reproductive diapause [3]. The molecular mechanisms of the JH signaling pathway regulating reproductive diapause have been better understood [4,5,6]. However, some evidence suggests that 20-hydroxyecdysone (20E) may also play a regulatory role in the reproductive diapause of insects. For example, levels of 20E in females were significantly lower during diapause in *Drosophila melanogaster* [7], *Locusta migratoria* [8], and *Protophormia terraenovae* [9]. Recently, Guo et al. have confirmed that 20E is critical for JH biosynthesis and reproductive diapause in *Colaphellus bowringi* [10]. Nevertheless, different results were also obtained. In Polygonia caureum, the 20E injection could not break diapause, and no difference was recorded in 20E titers of diapausing and non-diapausing adults, indicating no evidence of 20E involvement in diapause in this species [11]. Therefore, the regulatory role of 20E in reproductive diapause and its molecular mechanism are still necessary to be intensively investigated.

20E, the active form of the steroid hormone ecdysone, mediates gene expression after binding to a complex of two nuclear hormone receptors, the ecdysone receptor (EcR) and ultraspiracle (Usp), and its regulatory cascade includes several other nuclear hormone receptors (e.g., E75, E78, HR3, bFTZ-F1) and transcription factors (e.g., Broad (Br) and E74) [12]. Knockdown of *EcR* and *Usp* led to impairment of ovarian growth and maturation of the primary follicle in *Tribolium castaneum* [13]. In *Schistocerca gregaria*, silencing *EcR* depressed ovarian maturation and oviposition [14]. Knocking down *EcR* resulted in reduced fecundity in *Zeugodacus cucurbitae* [15], *Henosepilachna vigintioctopunctata*, and *Leptinotarsa decemlineata* [16]. *HR3*, an early 20E-responsive gene, is directly induced to express by 20E and follows the 20E titter during development [17]. BmHR3A, an isoform of HR3 of *Bombyx mori*, was accumulated in ovarian follicular cells during vitellogenesis, suggesting that BmHR3A may function as a regulator of vitellogenesis in the ovary [17]. The arrestment of ovary development is a key feature of reproductive diapause in insects. Therefore, we speculate that 20E may regulate reproductive diapause via EcR and HR3 in insects.

In this study, we investigated the role of 20E signaling in the regulation of reproductive diapause and its molecular mechanisms in *Galeruca daurica*, a new serious pest in the grasslands of Inner Mongolia, north China. *G. daurica* belongs to the Chrysomelidae family of Coleoptera and is an oligophagous pest that mainly damages the forage grasses of the genus *Allium* in the Liliaceae family, such as *Allium mongolium*, *A. ramosum*, and *A. polyrizum.* This leaf beetle occurs in one generation each year. The overwintering eggs begin to hatch in the middle of April. About one week after adult eclosion during summer, adults aggregate under cowpats, stones, and grass clusters, stop feeding, and turn into obligatory diapause. They finish diapause about three months later in autumn, then continue to feed and begin to mate [18,19]. Our previous studies have confirmed that JH signaling plays an important role in the regulation of reproductive diapause in *G. daurica* [5,6]. Here, we cloned and characterized the complete open reading frame (ORF) sequence of *EcR* from *G. daurica*. Injection of 20E stimulated the expression of *EcR*, *HR3*, and *vitellogenin* (*Vg*), whereas it suppressed the expression of *fat acid synthase* (*FAS*) and reduced the total lipid content, leading to delayed entry of diapause. Conversely, the knockdown of *EcR* or *HR3* repressed the expression of *Vg*, whereas it promoted the expression of *FAS* and increased total lipid content, resulting in advanced entry of diapause. These results indicate that 20E is involved in the regulation of reproductive diapause in *G. daurica* via a conserved EcR/HR3-dependent pathway.

## 2. Results

### 2.1. Cloning and Sequence Analysis of EcR in G. daurica

**Figure 1:**
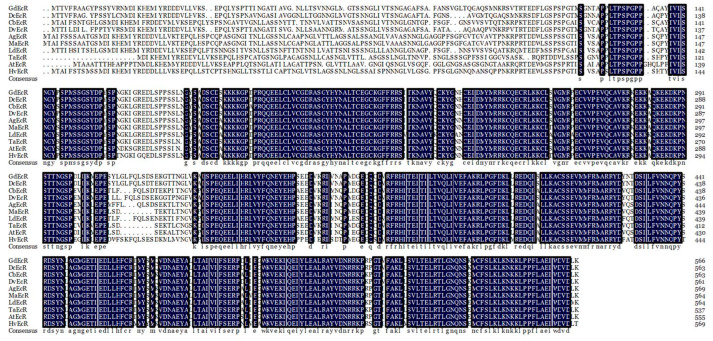
Multiple sequence alignments of the deduced amino acid sequence of *Galeruca daurica* ecdysone receptor (GdEcR) with other insect EcRs. Identical amino acids are shaded in black. Species abbreviations and GenBank accession numbers: DcEcR: *Diorhabda carinulata* (XP_057654933.1); CbEcR: *Colaphellus bowringi* (AHF52925.1); DvEcR: *Diabrotica virgifera virgifera* (XP_028128297.1); AgEcR: *Anoplophora glabripennis* (XP_018560850.1); MaEcR: *Monochamus alternatus* (AEY63780.1); LdEcR: *Leptinotarsa decemlineata* (QBH70333.1); TcEcR: *Tribolium castaneum* (KYB25531.1); ATEcR: *Aethina tumida* (XP_049823434.1); HvEcR: *Henosepilachna vigintioctopunctata* (BAP15926.1).

The complete ORF sequence of *GdEcR* (GenBank accession No.: OR637365) was cloned by RT-PCR from the *G. daurica* adults. The complete ORF sequence of *GdEcR* is 1704 bp in length, encoding 567 amino acids. The predicted protein sequence of GdEcR was aligned with orthologs from other species in Coleoptera. Sequence alignment revealed that GdEcR has the highest amino acid identity with *Diorhabda carinulata* DcEcR (95.09%), followed by *Diabrotica virgifera virgifera* DvEcR (90.83%), *Anoplophora glabripennis* AgEcR (75.61%), *Colaphellus bowringi* CbEcR (75.22%), *Aethina tumida* AtEcR (74.52%), *Monochamus alternatus* MaEcR (73.87%), *Tribolium castaneum* TcEcR (73.54%), *Leptinotarsa decemlineata* LdEcR (71.48%), and *Henosepilachna vigintioctopunctata* HvEcR (70.98%) (Figure 1).

### 2.2. Effects of 20E Treatment

**Figure 2:**
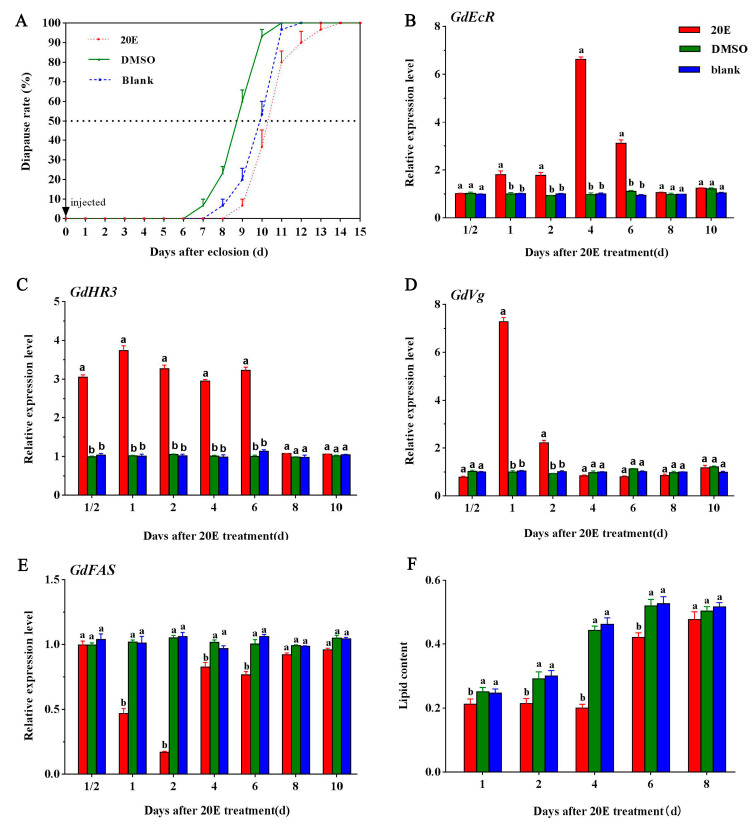
The effects of 20E treatment on diapause rate, expression level of diapause-related genes, and lipid content in *Galeruca daurica*. (**A**) Diapause rate; (**B**) The expression levels of *GdEcR*; (**C**) The expression levels of *GdHR3*; (**D**) The expression levels of *GdVg*; (**E**) The expression levels of *GdFAS*; (**F**) The total lipid content. Bars represent the means ± SE. Different letters indicate significant differences among different treatments by Duncan’s multiple range tests (*p* < 0.05). Blank means the blank control without treatment. Each treatment included three biological replicates, with 50 females per replicate for diapause observation and six females per replicate for gene expression, respectively.

To test whether 20E affects the diapause and expression of diapause-related genes in the *G. daurica* adult, we injected 2 μL of 20E (2.5 μg/μL) into the abdomen of a female adult 24 h post-ecolosion. The results showed that 20E treatment significantly affected adult diapause and the expression of diapause-related genes. The diapause rates of 20E treatment, solvent (DMSO) control, and blank control reached 50% on days 10.4, 8.69, and 9.57 post-eclosion, respectively (Figure 2A). Compared with DMSO control, the time for the diapause rate of 50% in 20E treatment was delayed by 1.71 days, indicating that 20E inhibited the *G. daurica* adults into diapause. Compared with DMSO control, the expression levels of *GdEcR* were significantly up-regulated by 0.78, 0.88, 4.96, and 2.17 times on days 1, 2, 4, and 6 post-20E treatment, respectively (Figure 2B), the expression levels of *GdHR3* were significantly up-regulated by 2.07, 2.66, 2.11, 190, and 2.21 times on days 0.5, 1, 2, 4, and 6 post-20E treatment, respectively (Figure 2C), and the expression levels of *GdVg* were significantly up-regulated by 7.20 and 3.26 times on days 1 and 2 post-20E treatment, respectively (Figure 2D). Conversely, the expression levels of *GdFAS* were significantly down-regulated by 56.44, 84.10, 18.56, and 23.62% on days 1, 2, 4, and 6 post-20E treatment, respectively (Figure 2E). In addition, total lipid content was significantly reduced from day 1 to 6 post-20E treatment (Figure 2F).

### 2.3. Effects of Silencing GdEcR/GdHR3 on the Expression of Diapause-Related Genes and Total Lipid Content

**Figure 3:**
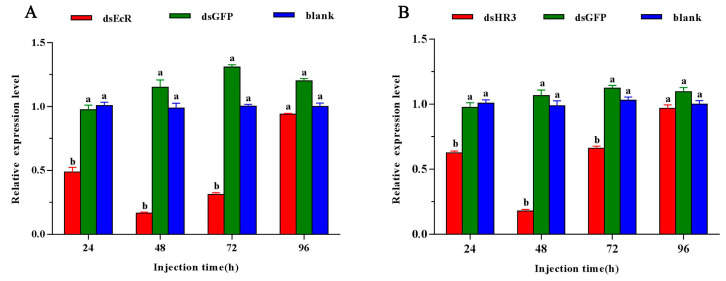
The efficiency of silencing *GdEcR* or *GdHR3* at 24, 48, 72, and 96 h after ds*GdEcR* or ds*GdHR3*-injection. (**A**) The efficiency of silencing *GdEcR*; (**B**) The efficiency of silencing *GdHR3.* Bars represent the means ± SE. Different letters indicate significant differences among different treatments by Duncan’s multiple range tests (*p* < 0.05). Blank means the blank control without treatment. Each treatment includes three biological replicates, with six females per replicate.

Female adults were collected on days 1, 2, 3, and 4 post-ds*GdEcR* or ds*GdHR3* injection, respectively, to further clarify the functions of *GdEcR* and *GdHR3* in reproductive diapause. The results showed that compared with those in the negative control (ds*GFP*), the expression levels of *GdEcR* reduced by 50.05, 85.69, 76.13, and 21.78%, respectively, on days 1, 2, 3, and 4 post-ds*GdEcR* injection (Figure 3A), and the expression levels of *GdHR3* reduced by 40.12, 62.92, 48.34, and 15.58%, respectively, on days 1, 2, 3, and 4 post-ds*GdEcR* injection (Figure 3B). The highest silencing efficiency was obtained on day 2 post-injection.

**Figure 4:**
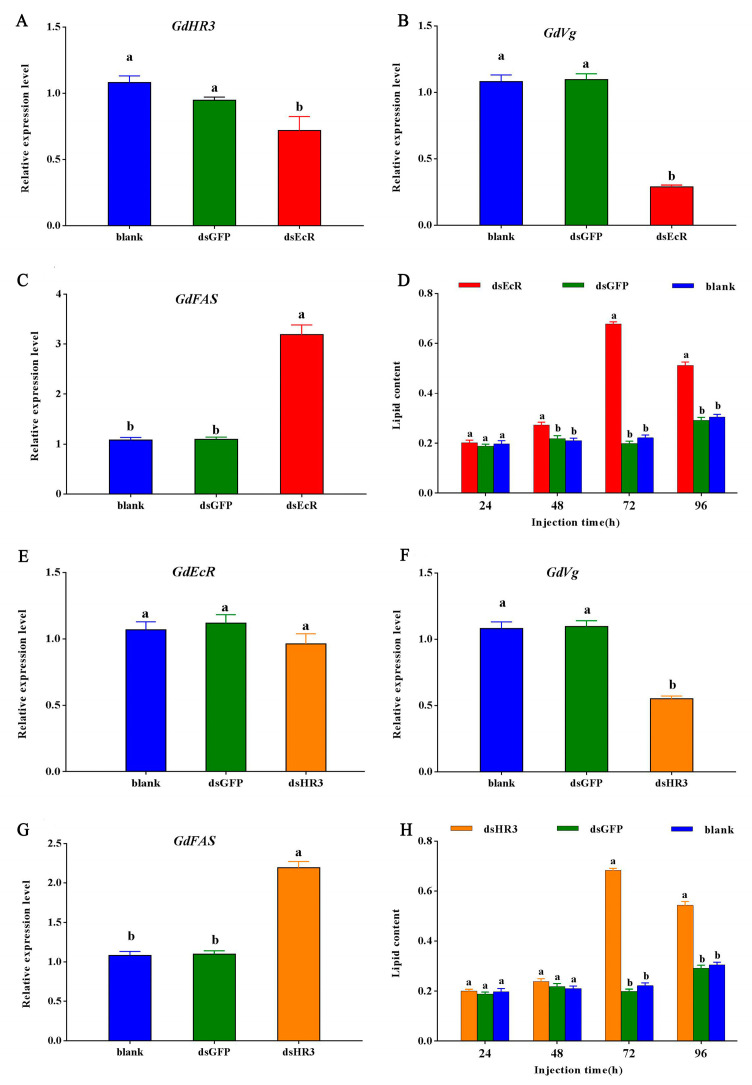
The effects of silencing *GdEcR* or *GdHR3* on expression levels of diapause-related genes at 48 h and total lipid contents at different times after ds*EcR* or ds*HR3*-injection. (**A**) The expression levels of *GdHR3* after silencing *GdEcR*; (**B**) The expression levels of *GdVg* after silencing *GdEcR*; (**C**) The expression levels of *GdFAS* after silencing *GdEcR*; (**D**) The total lipid contents after ds*EcR*-injection; (**E**) The expression levels of *GdEcR* after silencing *GdHR3*; (**F**) The expression levels of *GdVg* after silencing *GdHR3*; (**G**) The expression levels of *GdFAS* after silencing *GdHR3*; (**H**) The total lipid contents after ds*HR3*-injection. Bars represent the means ± SE. Different letters indicate significant differences among different treatments by Duncan’s multiple range tests (*p* < 0.05). Blank means the blank control without treatment. Each treatment included three biological replicates, with six females per replicate for gene expression and ten females per replicate for lipid assay, respectively.

Silencing *GdEcR* significantly decreased the expression levels of *GdHR3* (Figure 4A) and *GdVg* (Figure 4B), whereas it significantly increased the *GdFAS* expression (Figure 4C) and total lipid content (Figure 4D). Silencing *GdHR3* obtained the same results (Figure 4E–H).

### 2.4. Effect of Silencing GdEcR/GdHR3 on the Diapause of G. daurica Adults

**Figure 5:**
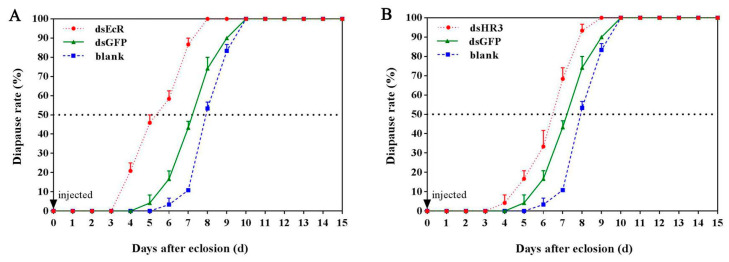
The effects of silencing *GdEcR* or *GdHR3* on diapause rate of adults *Galeruca daurica*. (**A**) The diapause rate of silencing *GdEcR*; (**B**) The diapause rate of silencing *GdHR3.* Blank means the blank control without treatment. Each treatment included three biological replicates, and each of the replicates contained 50 females.

The results showed that knockdown of *GdEcR* or *GdHR3* in 24 h post-eclosion significantly affected the development of adults, and the days for the diapause rate to reach 50% after ds*GdEcR* and ds*GdHR3* injection were 5.44 days and 6.49 days, respectively, whereas 7.22 days in the ds*GFP*-injected control (Figure 5A,B). Thus, the knockdown of *GdEcR* and *GdHR3* caused the adults to diapause for 1.78 days and 0.75 days ahead, respectively, indicating that reducing the expression of *GdEcR* and *GdHR3* induced reproductive diapause in *G. daurica*.

## 3. Discussion

The regulatory mechanism of insect diapause has been a focus on entomology. Recently, Guo et al. found that 20E signaling may play an important role in the regulation of reproductive diapause in *C. bowringi* [10]. However, *C. bowringi* is a species with long day-induced facultative diapause. It is unknown whether 20E signaling can mediate obligatory diapause. In this study, the application of 20E promoted the expression of *EcR* and *Vg*, whereas it repressed the expression of *FAS* and lipid accumulation, resulting in delayed entry of diapause in *G. daurica* adults. In contrast, knocking down *EcR* depressed the expression of *EcR* and *Vg*, whereas it induced the expression of *FAS* and lipid accumulation, causing earlier entry of diapause. Guo et al. also found that the application of 20E promoted vitellogenesis and ovarian development, whereas it inhibited fat accumulation in *C. bowringi* adults, and in contrast, silencing *EcR* stopped reproductive development and induced diapause traits [10]. Our previous studies showed that application of JH analogue (Methoprene) up-regulated *Vg* and down-regulated *FAS*, leading to repressed entry into diapause in *G. daurica* adults, and conversely, silencing JH receptor (*Met*) down-regulated *Vg* and up-regulated *FAS*, resulting in induced entry into diapause [5,6]. Liu et al. obtained similar results in *C. bowringi* [4]. These results indicate that both JH and 20E are involved in the regulation of reproductive diapause in insects. However, how 20E and JH harmoniously mediate reproductive diapause in insects remains poorly understood.

20E signaling pathway involves a series of genes, including *EcR*, *Usp*, *E75*, *E78*, *HR3*, *bFTZ-F1*, *Br-C*, and *E74*, which interact with each other. In this study, silencing *EcR* by RNAi repressed the expression of *HR3*, and the knockdown of *HR3* inhibited the expression of *EcR*. Similar results have also been reported. For instance, in *Blattella germanica*, the knockdown of *EcR* by RNAi reduced the expression of *E75A*, *E78*, and *HR3* [20]. Silencing *EcR* repressed the expression of *E74*, *E75*, and *HR3* in *Grapholita molesta* and *L. decemlineata* [21,22]. 20E signaling affects ovary development and lipid metabolism. In this study, silencing EcR or HR3 decreased the expression of *Vg*, whereas it increased the expression of *FAS* and total lipid content. In *L. decemlineata* and *H. vigintioctopunctata*, depletion of *EcR* by RNAi inhibited the transcription of *Vg* in the fat body [23]. In the *EcR*-knocked-down mutant of *Drosophila*, lipid accumulation was increased in the fat body [24]. Knockdown of *EcR* promoted lipid storage in *Aedes aegypti* [25]. These results indicate that 20E may regulate reproductive diapause in *G. daurica* via a conserved EcR/HR3 cascade.

## 4. Materials and Methods

### 4.1. Insects

On 8 May 2021, the larvae of *G. daurica* were collected from the Xilingguole grasslands of Inner Mongolia, China, and reared on *Allium mongolicum* in a chamber at 25 ± 1 °C under a 14L:10D photoperiod. Subsequently, the adults were selected for experiments.

### 4.2. RNA Extraction and cDNA Synthesis

Total RNA was extracted using the Trizol-based RNAios Plus reagent kit (TaKaRa, Dalian, China). The concentration was checked using a microspectrophotometer NanoPhotometer^®^ P330 (Implen, Munich, Germany) to ensure the quality of total RNA, and integrity was examined through electrophoresis in 1% agarose gel. cDNA was synthesized using the PrimeScript^®^ RT Reagent Kit with a gDNA Eraser (TaKaRa, Dalian, China).

### 4.3. Gene Cloning and Sequence Analysis

On the basis of the transcriptome database (PRJNA471603) that was assembled in our lab, the cDNA of *GdEcR* was amplified using the specific primer pairs (Table S1). The PCR procedure was set as follows: initial denaturation at 94 °C for 3 min. Subsequently, 35 cycles of 94 °C for 40 s were performed, primer annealing at the appropriate annealing temperature for 30 s, and extension at 72 °C for 90 s. After that, a final extension was conducted at 72 °C for 10 min. The PCR products were ligated to the pMD-19T vector (TaKaRa, Dalian, China) and transformed into *Escherichia coli* DH5α for cultivation. The open reading frame (ORF) was confirmed after sequencing alignment. The amino acid sequence was analyzed using the National Center for Biotechnology Information (NCBI) BLAST “https://blast.ncbi.nlm.nih.gov/Blast.cgi (accessed on 10 July 2021)”.

### 4.4. Quantitative Real-Time PCR Experiment (qRT-PCR)

qRT-PCR analysis was performed using FTC-3000P Real-Time Quantitative Thermal Cycler systems (Funglyn Biotech, Toronto, ON, Canada). The primers were designed using the NCBI online tool “http://www.ncbi.nlm.nih.gov/tools/primer-blast/ (accessed on 25 July 2021)” listed in Table S1. The reaction was performed in a total volume of 20 μL, which was composed of 2 μL of cDNA, 10 μL of GoTaq^®^ qPCR Master Mix, 0.4 μL of primer (with a concentration of 10 µmol/L), and 7.2 μL of ddH_2_O. The qRT-PCR reaction program was set as follows: the reaction mixture was incubated at 95 °C for 10 min. Subsequently, 40 cycles were carried out, with each cycle encompassing three steps: first, denaturation at 95 °C for 20 s; second, annealing at 60 °C for 90 s; and third, extension at 72 °C for 30 s. Following that, a melting curve analysis was performed. The analysis of relative expression levels was carried out using the 2^−∆∆Ct^ method [26]. The succinate dehydrogenase (*SDHA*) was used as a reference gene [27], and target gene expression was used as the control in the DMSO or dsGFP (green fluorescent protein) treatment for the ddCT analysis. Three biological replicates with six females per replicate and four or five technical replicates per biological replicate were performed in the qRT-PCR experiments.

### 4.5. 20E Treatment

To elucidate the impacts of 20E (purchased from MedChemExpress, Shanghai, China) on the expression levels of *GdEcR*, *GdVg*, and *GdFAS*, we first diluted 20E to a concentration of 25 mg/mL with dimethyl sulfoxide (DMSO) as stock solution. The 100 μL stock solution was diluted with 900 μL saline into 2.5 μg/mg work solution. The 2 μL 20E work solution was injected into the abdomen of a female adult 24 h post-eclosion, with a female adult injected with DMSO diluted with saline used as a negative control and a non-injected female adult as a blank control. Insects were reared on *A. mongolicum*, and collected for qRT-PCR analysis at 0.5, 1, 2, 4, 6, 8, and 10 d after the treatment. Moreover, three biological replicates were carried out for each treatment, with six female insects included in each replicate.

### 4.6. RNA Interference (RNAi) Experiments

Firstly, *GdEcR* and *GdHR3* were amplified by means of special primers that incorporated the T7 promoter sequence (as shown in Table S1). Subsequently, the PCR products were cloned into the pGEM^®^-T Easy vector (Promega, Beijing, China) and then transformed into DH5α competent cells for the purpose of direct sequencing. Double-stranded RNAs (dsRNAs) targeting *GdEcR* and *GdHR3* were synthesized using the T7 RiboMAX™ Express RNAi System kit (Promega, Beijing, China), following the instructions provided by the manufacturer precisely. The quality of *GdEcR* and *GdHR3*-dsRNA was checked by agarose gel electrophoresis (1%) and a Nano PhotometerTMP-Class (Implen, Munich, Germany). After that, ds*GdEcR* or ds*GdHR3* was dissolved in enzyme-free water to a concentration of 1000 ng/μL and then stored in a freezer maintained at −80 °C.

Two μL of ds*GdEcR* or ds*GdHR3* (1000 ng/μL) were injected into the body of the test insects through their abdomens with a microinjector (SHIMADZU, Kyoto, Japanv). dsRNA for *GFP* (dsGFP) was produced and used as a negative control, and untreated females as a blank control. Three biological replicates were conducted, with 50 females per replicate. The efficiency of RNAi was evaluated at 24, 48, 72, and 96 h following injection. Furthermore, the impact of RNAi on the expression levels of *GdEcR*, *GdHR3*, *GdVg*, and *GdFAS* were examined through qRT-PCR. In addition, the total lipid content was determined after injection, and the diapause situation was also observed.

### 4.7. Measurement of Total Lipid Content

The determination of total lipid content was carried out in accordance with the chloroform-methyl alcohol method [28]. The procedure was as follows: Firstly, the treated insects were placed in a centrifuge tube and then dried in an oven maintained at 60 °C for 3 days. Subsequently, their dry mass (DM) was measured by weighing. After that, the dried insects were ground in 1 mL of a chloroform-methanol (2:1) solution. Following grinding, the mixture was subjected to centrifugation. The lean dry mass (LDM) was measured after being placed in an oven at 60 °C and baked until it reached a constant weight. Total lipid content (FC) was calculated using the formula FC = (DM − LDM)/LDM. For each treatment, three independent biological replicates were performed. In each replicate, 10 female insects were included, and one individual was assayed each time to ensure the accuracy and reliability of the results.

### 4.8. Developmental Observation

Females were observed twice daily to monitor their feeding behavior, and the number of females that were feeding was recorded on a daily basis after the treatment. Diapause entry was determined when females began to cease feeding continuously. This was based on our previous observations, which demonstrated that adults typically started to stop feeding approximately one week after eclosion and then entered diapause, accompanied by a sudden decline in respiration intensity [18,19]. Each treatment comprised three biological replicates, with each replicate consisting of 50 females.

### 4.9. Statistical Analysis

The data presented in this study are expressed as mean ± standard error (SE). Statistical significance was determined using SPSS 20.0 software. Significant differences were determined through one-way ANOVA, followed by Duncan’s multiple-range tests (*p* < 0.05).

## 5. Conclusions

**Figure 6:**
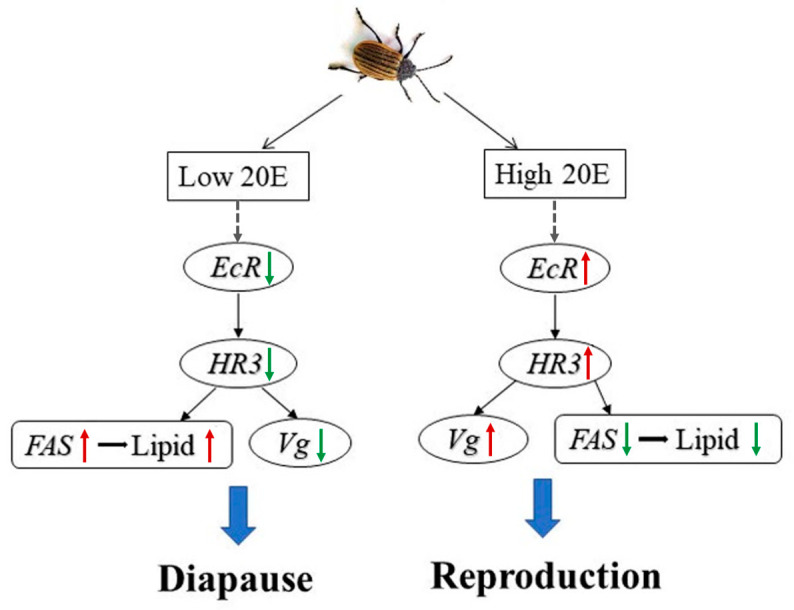
A model for 20E signaling in reproduction and diapause of adult *Galeruca daurica*. 20E signaling induces reproduction by promoting *Vg* expression via a conserved EcR/HR3 cascade. Females reduce lipid accumulation due to *FAS* repression. The block of 20E signaling via Met/HR3 induces reproductive diapause by inhibiting *Vg* expression. Females increase lipid accumulation due to *FAS* promotion. Therefore, 20E-EcR-HR3 signaling facilitates the antagonism between reproduction and diapause in *Galeruca daurica*. The green arrows indicate down-regulation and the red arrows indicate the up-regulation, respectively.

This study indicates that 20E may regulate reproductive diapause in *G. daurica* via a conserved EcR/HR3-dependent pathway. A mode is proposed to describe the mechanism of antagonism (Figure 6). 20E inhibits lipid accumulation by down-regulating *FAS* via a conserved *EcR/HR3* cascade while promoting *Vg* expression to repress reproductive diapause. Conversely, the block of 20E signaling via *EcR/HR3* increases lipid accumulation by up-regulating *FAS* while reducing *Vg* expression to induce reproductive diapause.

## Data Availability

The data sets supporting the results are included within the article.

## Supplementary Materials

The following supporting information can be downloaded at: https://www.mdpi.com/article/10.3390/ijms252312976/s1.
