# Supplementary material for: 20-Hydroxyecdysone Mediates Reproductive Diapause in Galeruca daurica via Ecdysone Receptor EcR and Nuclear Hormone Receptor HR3
Source: Int J Mol Sci. 2024 Dec 3;25(23):12976. doi: 10.3390/ijms252312976 (PMC11641180; doi:10.3390/ijms252312976)
Supplement: Supplementary file 1 [file ijms-25-12976-s001.zip › ijms-3328201-supplementary.pdf]

Table S1 The special primers used in this study.

Genes	Forward primers (5' to 3')	Reverse primers (5' to 3')
**Gene-cloning**		
EcR	AAATATTACACTTAGAAACACCGCC	GAACAACTTATCTATGAGCAGATGG
**qRT-PCR**		
EcR	CTACACATTCCGGCCTCATT	AGGACCAACACCTCGACAAC
HR3	GAAAGCGTCTTGGGGTAACA	CGATTGCGCACAGAAACTTA
Vg	TTGGTTGAACAGCAGCTTTG	GGACACTGTTTTGCCCCTAA
FAS	GAAGCGTTATGGTCCCATGT	GAACAAGACTGCCGAGAACC
SDHA	GGGAGACCACCATCTCCTCA	AGCTGGTGCTCCTAAGTCCA
**RNAi**		
EcR	TAATACGACTCACTATAGGGGCGCAC<br>CGAAGACTTTTTAG	TAATACGACTCACTATAGGGCGTGAGG<br>GCGTTATAGTGGT
HR3	TAATACGACTCACTATAGGGCAAACC<br>CCACGATCTCTCAC	TAATACGACTCACTATAGGGGCTATGCT<br>TTTCGCCACTTC
GFP	TAATACGACTCACTATAGGGCATAGG<br>CCACACTTGTCACTACTTT	TAATACGACTCACTATAGGGGTGGACA<br>GGTAATGGTTGTCTGGTA

The underlined part is the T7 promoter sequence.
